# Eggshell Membrane as a Biomaterial for Bone Regeneration

**DOI:** 10.3390/polym15061342

**Published:** 2023-03-08

**Authors:** Adriana Torres-Mansilla, Maxwell Hincke, Ana Voltes, Elena López-Ruiz, Paula Alejandra Baldión, Juan Antonio Marchal, Pedro Álvarez-Lloret, Jaime Gómez-Morales

**Affiliations:** 1 Departamento de Geología, Universidad de Oviedo, 33005 Asturias, Spain; 2 Department of Innovation in Medical Education, Faculty of Medicine, University of Ottawa, Ottawa, ON K1H8M5, Canada; 3 Department of Cellular and Molecular Medicine, University of Ottawa, Ottawa, ON K1H8M5, Canada; 4 Biopathology and Regenerative Medicine Institute (IBIMER), Centre for Biomedical Research (CIBM), University of Granada, 180171 Granada, Spain; 5 Instituto de Investigación Biosanitaria ibs. Granada, University Hospitals of Granada–University of Granada, 18071 Granada, Spain; 6 BioFab i3D Lab–Biofabrication and 3D (bio)Printing Singular Laboratory, Centre for Biomedical Research (CIBM), University of Granada, 180171 Granada, Spain; 7 Department of Health Sciences, Campus de las Lagunillas S/N, University of Jaén, 23071 Jaén, Spain; 8 Departamento de Salud Oral, Facultad de Odontología, Universidad Nacional de Colombia, Bogotá 111321, Colombia; 9 Laboratorio de Estudios Cristalográficos IACT–CSIC–UGR, Avda. Las Palmeras, No. 4, Armilla, 18100 Granada, Spain

**Keywords:** biopolymer, bone biomaterial, bone scaffold, bone tissue engineering, eggshell membrane, organic matrix

## Abstract

The physicochemical features of the avian eggshell membrane play an essential role in the process of calcium carbonate deposition during shell mineralization, giving rise to a porous mineralized tissue with remarkable mechanical properties and biological functions. The membrane could be useful by itself or as a bi-dimensional scaffold to build future bone-regenerative materials. This review focuses on the biological, physical, and mechanical properties of the eggshell membrane that could be useful for that purpose. Due to its low cost and wide availability as a waste byproduct of the egg processing industry, repurposing the eggshell membrane for bone bio-material manufacturing fulfills the principles of a circular economy. In addition, eggshell membrane particles have has the potential to be used as bio-ink for 3D printing of tailored implantable scaffolds. Herein, a literature review was conducted to ascertain the degree to which the properties of the eggshell membrane satisfy the requirements for the development of bone scaffolds. In principle, it is biocompatible and non-cytotoxic, and induces proliferation and differentiation of different cell types. Moreover, when implanted in animal models, it elicits a mild inflammatory response and displays characteristics of stability and biodegradability. Furthermore, the eggshell membrane possesses a mechanical viscoelastic behavior comparable to other collagen-based systems. Overall, the biological, physical, and mechanical features of the eggshell membrane, which can be further tuned and improved, make this natural polymer suitable as a basic component for developing new bone graft materials.

## 1. Introduction

The avian eggshell membrane (ESM) is a versatile biomaterial with chemical characteristics and structural properties that could be exploited for bone regeneration [1]. It contains molecules of biomedical interest, such as collagen, hyaluronic acid, and dermatan sulfate [1]. Moreover, it has a mesh-type structure that behaves mechanically similarly to collagenous systems, such as tendons [1,2]. These properties resemble those of the collagenous matrix of bone, making the ESM a potential basis for bone scaffold development. Moreover, research for this ESM application constitutes an emerging subject that could be further amplified.

There is an ongoing demand for novel materials for bone regeneration due to the high prevalence of bone diseases. These diseases can lead to severe bone abnormalities and bone fragility, which in turn cause disability and decrease the quality of life [3,4,5,6]. Moreover, traumatic injury can require the replacement of damaged bone [7]. Currently, the most suitable material for bone regeneration is autologous bone. However, there are disadvantages and limitations regarding its extraction and possible post-operative complications after harvest [8,9]. Therefore, exploring the development of bone biomaterials with microstructure, biocompatibility, and bone-forming ability that overcome the limitations of autologous bone constitutes an active field of research [8]. Moreover, it is also essential to consider the cost-effectiveness of a biomaterial, which could lower the economic burden of high-priced biomedical materials [10].

Bone scaffolds are composite materials that can be fabricated using polymers, ceramics, or metals [11], as typified by a recently developed magnesium-based scaffold [12]. Polymers constitute reliable and versatile materials for fabricating bone scaffolds, primarily due to their broad biodegradation tunability, surface-to-volume ratio, heterogeneous porosity, and mechanical characteristics [13,14,15,16]. Polymeric materials also have considerable design potential arising from simple customization of their chemical and structural properties. Polymers can be synthetic or natural. Synthetic polymers have predictable properties and controllable synthesis [13,14]. However, they usually lack cell adhesion sites and are derived from nonrenewable resources [14,16]. Therefore, the sustainability of synthetic polymers is a potential drawback [16]. Some examples of these kinds of polymers are aliphatic polyesters, including poly-ε-caprolactone (PCL) and polylactic-based polyesters (PDLA, PLLA) [15]. Natural polymers are more sustainable and biodegradable than synthetic materials [13,14,16]. They possess molecules that are biomimetic, promote bioactivity, and support bone remodeling [15]. Consequently, it is worthwhile to investigate the properties of natural polymers to create bone biomaterials.

Moreover, biomaterials for bone regeneration based on natural polymers have great promise, since they are sustainable and contribute to a circular economy [17]. Biopolymers, notably cellulose, chitosan, and alginate, have been used as bone biomaterials [13,18,19]; however, they present shortcomings, including insufficient degradation [20], the presence of impurities [21], and limited long-term stability in physiological conditions [22]. The biodegradability, stability, and low immunogenicity of the ESM could compensate for the shortcomings of other biopolymers [23]. In addition, reusing this membrane requires little manufacturing, which exploits the advantages of its unique biological and mechanical properties [24]. Finally, research on the ESM for bone tissue engineering shows considerable potential due to the relative lack of studies associated with this application.

The mechanical and biological properties of ESM, as well as the low economic value of the readily available chicken eggshell membrane, make it a suitable starting material for bone regeneration material development. This paper presents a brief overview of the requirements for a bone biomaterial scaffold, followed by a summary of ESM extraction processes, as well as a description of its main chemical characteristics and mechanical and biological properties, all intended as a guide for the reader. This review of these aspects provides a coherent context for understanding the suitable characteristics of the ESM, which encourages its use for bone regeneration biomaterial development.

Recent literature reviews on the features and characteristics of ESM have underlined its importance for different technological and industrial applications [1,24,25,26,27,28,29,30]. However, a compilation of its biological, physicochemical, and mechanical properties that highlight the ESM’s value as a candidate for bone regeneration material has not yet been performed. The current review summarizes the state-of-the-art literature of these ESM properties and identifies gaps and needs in the path toward its exploitation as a new biomaterial for development in bone tissue engineering.

## 2. Criteria for the Development of Bone Scaffolds

**Figure 1:**
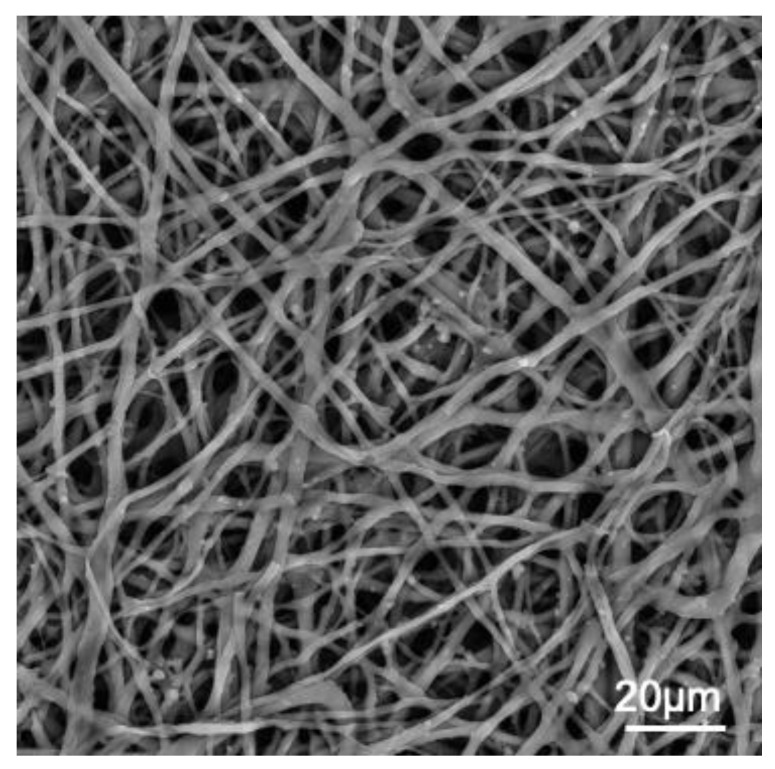
SEM image of chicken ESM. Photo credit: G. Kulshreshtha and M. Hincke (unpublished).

The ESM (Figure 1) possesses microstructural and compositional characteristics that suggest this matrix could be a promising candidate for developing scaffolds for bone regeneration. A scaffold is a biomaterial with a three-dimensional structure that provides an appropriate environment for bone-building cells. Its goal is to replicate the collagen/apatite extracellular matrix of the osseous tissue and its innate functions, so that the behavior of incoming cells will mimic their native state [31]. The structure and composition of a scaffold should stimulate cellular attachment, proliferation, and differentiation, promoting the regeneration of bone tissue [32,33,34]. Ideally, these materials should mimic the natural bone matrix [34]. However, due to the intricacy of the chemical and microstructural makeup of bone, this work touches upon numerous research domains. Bone is a highly hierarchical biohybrid material comprising an organic matrix made primarily of collagen and an inorganic phase composed of apatite [34]. The basic building block of the bone microstructure is a self-assembled collagen fibril mineralized with apatite nanocrystals [35,36,37,38]. Bone apatite is also a highly ionic-substituted reactive mineral with specific crystallographic properties and coated with citrate molecules [39,40,41,42,43]. 

**Figure 2:**
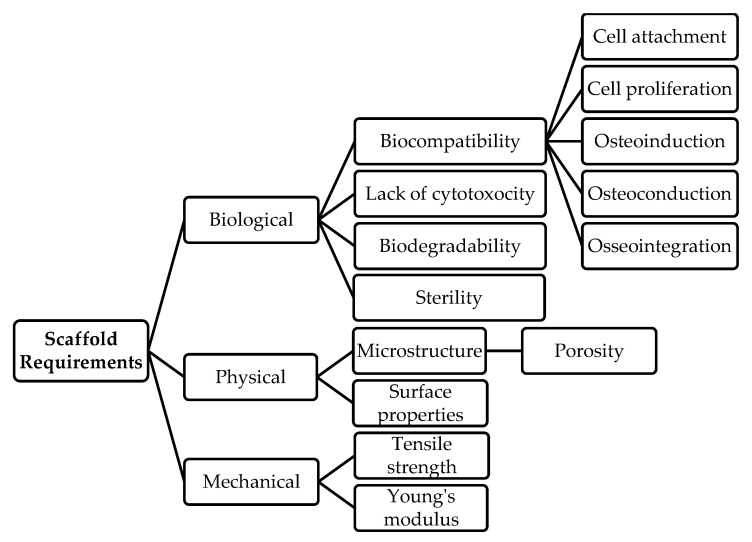
Scaffold requirements, from references [32,33,34].

Bone possesses a number of simultaneous properties: it is organic, inorganic, reactive, stable, strong, and flexible. Moreover, this mineralized tissue presents an architecture that is hierarchically organized with a gradient structure and displays anisotropic properties [44]. It is a structure sculpted through adaptative processes whose mechanical properties support vital physiological functions, such as movement and protection of internal organs [45]. These features present, in turn, a challenge for material design. Nevertheless, previous research has evaluated certain characteristics that scaffolds must possess to achieve good quality bone regeneration [32,33,34]. These interdependent characteristics can be described as the biological, physical, and mechanical requirements that are summarized in Figure 2.

Briefly, the biological requirements comprise biocompatibility, lack of cytotoxicity, biodegradability, and stability during sterilization procedures. Biocompatibility is the ability of an implanted material to coexist and perform cohesively with the surrounding tissue without side effects such as cytotoxicity, carcinogenesis, mutagenesis, genotoxicity, and immunogenicity [46]. Biocompatibility encompasses good cell attachment and proliferation and osteoinductivity [32,33,34]. The property of osteoinductivity means that the material can induce cells to differentiate into osteogenic cells. For example, the material should induce the differentiation of mesenchymal stromal cells (MSCs), bone-marrow-derived stromal cells (BMSCs), and adipose-derived stromal cells (ADSCs) into osteoblasts [33]. Osteoconductivity describes the ability of the osteogenic cells and their byproducts to migrate into the scaffold and replace it with new bone (e.g., stimulates angiogenesis) [34]. Another significant feature of bone scaffolds and other implantable materials is osseointegration. This means that the scaffold and bone integrate so that only a fracture can separate the scaffold material from the newly regenerated bone. This property is related to the physical properties of the scaffold, its microstructure (e.g., porosity), and its surface properties (namely topography and surface chemistry) [47,48].

In addition to promoting osteogenesis, bone scaffold materials should not display negative effects that could damage healthy local tissue, implying the absence of cytotoxicity [34]. Implanted biomaterials will inevitably cause a foreign body reaction. Still, they should exhibit minimal inflammatory or immunological reactions and not create any harmful byproducts at the site of implantation in the host [32,33,34]. The scaffold only functions as a temporary structure within the regenerating tissue, so it should disintegrate over time [34]. Therefore, biodegradability constitutes a key biological aspect for a safe and programmed replacement of the biomaterial as it transforms into bone. This property must comply with a balance between biomaterial decomposition and new bone formation. While the biomaterial must break down so that future invasive surgery is unnecessary [34], it should maintain stability and degrade without releasing harmful by-products. Lastly, because the bone biomaterial will be implanted into the body, it must be sterilizable in a way that preserves its primary qualities [33].

Ideally, the scaffold should possess physical and mechanical characteristics reminiscent of the bone organic mineralized matrix structure, with a suitable architecture that provides stability without loss of bioactivity [34]. In this regard, the microstructure is a fundamental aspect of tuning and optimizing the functional and mechanical needs of a scaffold for bone tissue engineering [49]. One of the most revisited aspects is porosity, which allows cell migration, angiogenesis, and transport of nutrients and waste. The quantity and size and shape of pores and pore connectivity should be enough to provide these characteristics without compromising the structure of the scaffold [32,33,34]. With respect to porosity, it is essential to also take into account the tortuosity of the material, as this can influence the permeability of the scaffold and provide better cell attachment compared to scaffolds made of relatively straight microchannels. In the design of bone scaffolds, tortuosity must be modeled to obtain permeability values comparable to those present in the bone structure [50]. Other surface properties related to the microstructure (e.g., hydrophilicity, topography, and surface chemistry) will also influence the biocompatibility of the material [47,48,51].

The microstructure of the biomaterial influences its mechanical properties, usually described in terms of tensile strength and Young’s modulus, among other characteristics (Figure 2). Changes in bone microstructure related to age and disease, for instance, increased porosity, hypermineralization, and damage accumulation, are associated with decreased bone strength [52]. Therefore, tailoring the scaffold with a resistant and biocompatible structure is crucial for bone tissue engineering [53]. The creation of the scaffold also needs to be commercially affordable for clinical viability. Manufacturing of the scaffold should be both economical and industrially scalable [54]. 

The ESM is a biomaterial that potentially satisfies most of the bone scaffold requirements. As discussed in detail in the following sections, the ESM demonstrates favorable outcomes during in vitro and in vivo testing, which confirms its biocompatibility. The ESM induces the attachment, proliferation, and differentiation of various cell types. Additionally, ESM implanted in bone and periodontal defects is observed to encourage regeneration [23,55,56,57]. All of these outcomes for in vivo implantation are associated with a low proportion of dead cells and minimal inflammation, demonstrating the absence of cytotoxicity [23,55,56,57,58,59]. Moreover, the ESM has an extended time span for biodegradability [23,54] and can be sterilized prior to implantation [55,60,61].

Regarding its physical and mechanical characteristics, the ESM possesses a layered, mesh-like, and fibrous proteinaceous microstructure that is highly insoluble but customizable [1,29]. Moreover, the ESM is porous and hydrophilic, and its surface properties can be adjusted to enhance biocompatibility. For example, the ESM microstructure has been modified by treatment with citric acid [58,59], decoration with carbon nanodots [62], and cross-linking with natural and synthetic polymers [63,64,65]. These structural modifications enhance its biocompatibility, and in some cases, also enhance its antibacterial activity [58]. Associated with these structural and surface properties, it has been observed that the ESM exhibits viscoelastic mechanical behavior [2]. Thus, the ESM resists environmental impacts by transmitting, dissipating, and storing force and energy comparable to a collagen matrix system [2]. This mechanical behavior is noteworthy considering the possibility of employing the ESM to replicate the collagen/apatite extracellular structure of bone. Therefore, the biological, physical, and mechanical properties of the ESM meet most of the requirements needed for a bone scaffold. In addition, the ESM can be chemically modified for the development of novel materials for bone regeneration. It is essential to add that exploitation of ESM for biomaterial science constitutes the reuse of a resource generally considered to be a waste product of the egg-breaking industry [1,17].

## 3. Membrane Extraction

**Figure 3:**
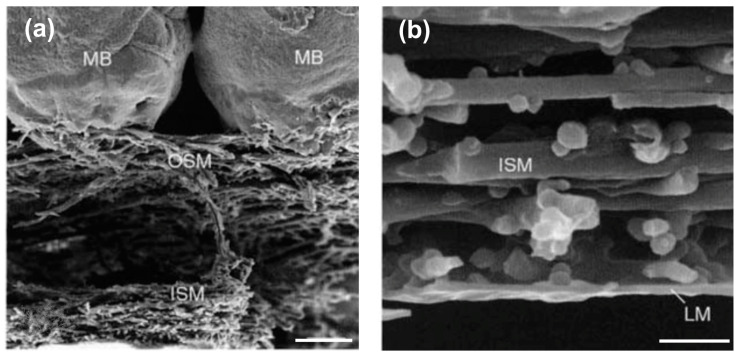

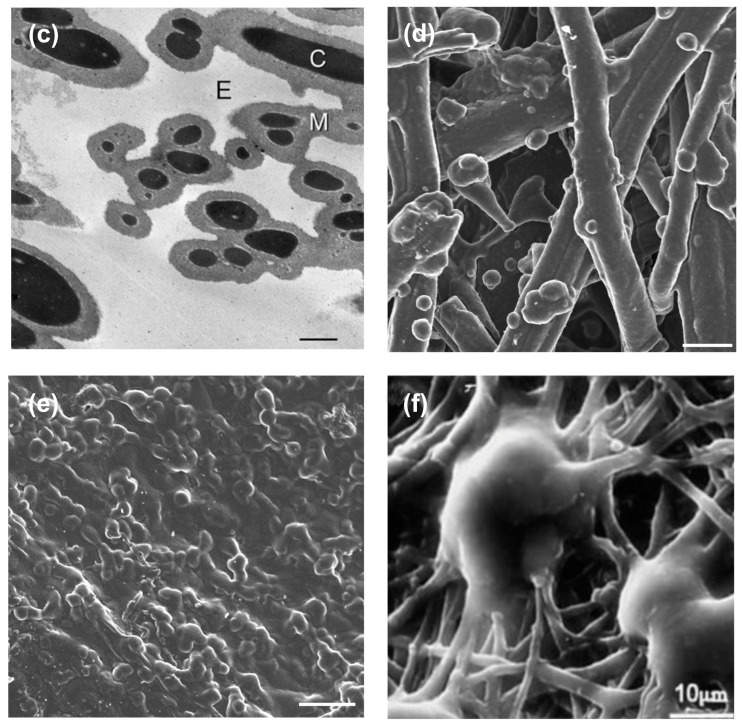
Eggshell membrane structure. (**a**) SEM micrograph of membrane and mammillary columns/bodies interface. It is possible to differentiate the inner shell membrane (ISM) from the outer shell membrane (OSM). The mammillary bodies (MB) are also marked, scale bar 20 μm. (**b**) Detail of the inner membrane (ISM) and the limiting membrane (LM), scale bar 2 μm. Figures (**a**,**b**) are reproduced from Hincke et al. [95]. Copyright from ELSEVIER (License number 5443170652149). (**c**) TEM micrograph of outer membrane fibers, depicting the highly electron-dense collagen-containing core (C) and the less electron-dense glycoproteic mantle (M), separated by extra-fiber spaces (E) Figure reproduced from Li et al. [96] Copyright ELSEVIER (License number 5443170450243). (**d**) View from the outer membrane surface, scale bar 2 μm. Original images. (**e**) View from the inner surface of the shell membranes, scale bar 4 μm. Original images. (**f**) Mammillary knobs before calcification], scale bar ∼10 μm. Figure reproduced from Arias et al. [97] Copyright WILEY-VCH Verlag GmbH & Co. KGaA (License number 5443171122915).

**Figure 4:**
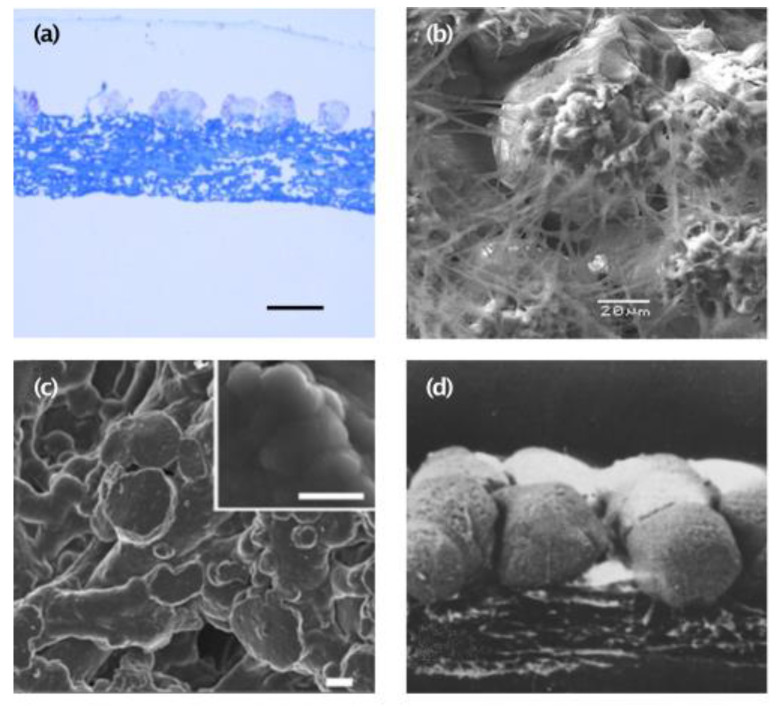
(**a**) Optical microscopy photography collected seven hours post ovulation (p.o.). Mineralization begins immediately after the pseudo-periodic deposition of rounded organic-rich structures (5 h p.o.) on the outer membrane surface fibers, scale bar 50 μm. Image reproduced from [67] Copyright Elsevier (License number 5443200070329). (**b**) SEM micrography shows the connection between the fibers and the mammillary cones, scale bar 20 μm. Image reproduced from [66]. Copyright Elsevier (License number 54432000406930). (**c**) Initial formation of flat disc-shaped ACC particles on the membrane fibers, scale bar 1 μm. Image reproduced from [67] Copyright Elsevier (License number 5443200070329). (**d**) SEM micrography of eggshell obtained by sodium chlorate treatment with short, spherical, non-fused, spherical columns, zoom 240x. Image reproduced from [103]—copyright Elsevier (license number 5443200566496).

The individual fibers of the outer ESM penetrate and are embedded into the tips of the mammillary cones (Figure 3a and Figure 4b). Thus, the ESM is tightly bound to the eggshell through a complex transitional structure in which the mammillary knobs are integrated into the mineral mammillary columns, which are continuous into the crystalline palisade region that contributes most of the eggshell thickness [66,67,68]. This strong adhesion makes the difficulty in obtaining large, intact pieces of the ESM a factor to consider in obtaining this biomaterial [1]. In bench-scale experimentation, ESM separation is usually performed by manual peeling [2,23,58,59,64,69,70,71], chemical dissolution of the eggshell mineral [72], or enzymatic treatment. However, this last method is mainly used to obtain hyaluronic acid from isolated ESM [73]. For manual separation, the membrane can be obtained largely intact, although mechanical extraction may affect the transient attachment structures [29]. Moreover, fibers of the outer membranes remain attached to the mineral and are not present in the purified membranes.

Commonly, dissolution of the eggshell calcitic mineral has been achieved with acetic acid (CH_3_COOH), hydrochloric acid (HCl), or ethylenediaminetetraacetic acid (EDTA) [65,74,75,76,77]. Other less commonly used chemicals, e.g., n-butyl acetate, have been considered [74]. Chemical treatment affects protein bonds and has an impact on the structural integrity and chemical properties of the membrane [78]. In addition, previously published research has not established the minimum solution concentration, time, and temperature to dissolve a given amount of eggshell. As a result, there is an extensive range of these variables reported in the literature (Table 1). The calcium reserve body (CRB) at the base of the mammillary cone is selectively demineralized during such treatment, so fragments of the mammillary cone tips may remain attached to the resulting sheets of ESM, requiring additional acid treatment to dissolve adhering calcium carbonate (CaCO_3_) [79].

A variety of separation methods have been developed for the large-scale industrialized purification of ESM from eggshell [80] (Table 2). These include microwave-assisted membrane detachment [81], flash evaporation [82], and dissolved air flotation [83]. Microwave-assisted separation consists of a weakening of the physical bonds between membranes and the eggshell. Since the membranes contain more water than the eggshells, the two components heat up differentially. The membranes absorb more energy from electromagnetic waves, expand, and separate from the eggshell [81,84]. Flash evaporation uses a batch reactor that takes advantage of the effect of pressure changes of a saturated liquid to separate the membrane. Chi et al. reported a separation rate of ~69% [82]. Lastly, dissolved air flotation separates the membrane from the shell according to its differential density by applying a water flow and mixed air. The disadvantage of this method is that the unseparated shell must be crushed entirely before separation [83].

Several devices have been developed and patented to optimize ESM separation on an industrial scale [80]. Generally, these machines break or pulverize the eggshell using, among other methods, airflow [85], cavitation [86], or the Venturi effect [87] to separate the flexible ESM. Several additional patented approaches use mechanical separation systems [88,89,90]. The impact of these separation techniques on the chemical and structural integrity of the membrane, however, must be considered with respect to the ultimate application and is a fertile field of investigation. 

Recent reviews of ESM separation methods have described their advantages and limitations [1,24,25,26,29,30,74,80]. It is important to emphasize that an ideal process that effectively retrieves large pieces of the membrane with minimal alteration is still a matter of active study [1]. The ESM consists of valuable molecules, including collagens and hyaluronic acid, in addition to large numbers of proteins [91], which contribute to its fibrous biopolymeric nature with viscoelastic properties [92]. Suitable recovery of the ESM would allow these chemical and structural properties to be exploited and applied to develop materials for bone regeneration.

## 4. Eggshell Membrane Structure and Composition

The forming egg acquires its constituents as it passes through specialized regions of the avian oviduct [72]. In the white isthmus region, the tubular gland cells secrete the precursors of the ESM. The resulting fibers assemble into membranes that surround the rotating immature, uncalcified egg white while it traverses this region [93]. The membrane is progressively deposited as an interlaced fiber meshwork with three morphologically distinct layers: a thin limiting membrane, an inner membrane, and an outer-layer membrane (Figure 3a,b,d,e) [94]. 

The limiting membrane consists of a very thin structure only a few microns in thickness, which surrounds the egg white (Figure 3b) and functions as a barrier to restrict the leakage of egg white and yolk [76]. Fluorescein isothiocyanate (FITC) staining reveals that most spaces within the inner membrane and a large portion of its width are filled by the limiting membrane [94]. The fibers of the outer and the inner membranes are interlaced throughout most of their surface but become separated at the air cell (broad end of the egg). Each fiber presents a similar construction, with a core rich in collagen, surrounded by a fuzzy glycoproteic mantle [96,98]. However, the fiber position, orientation, and size differ for each membrane layer. The inner membrane is thinner than the outer membrane, being ~15–26 μm thick, with a smaller fiber width of 0.1 to 3 μm and a diameter of 1.5 to 2 μm [1,66,94,99]. The outer membrane is ~50–70 μm thick, with fibers 1 to 7 μm in width and 2.5–5 μm in diameter. The fibers of the outer ESM penetrate the mammillary knobs of the shell, forming a bud-like structure that is partially calcified (Figure 3a,d) [1,66,94,99]. Overall, the total membrane thickness is approximately 100 μm [92].

The outer membrane has distinctive structures on its outer surface named mammillary knobs (Figure 3f). These are discrete organic matter aggregations that function as nucleation sites for calcite. These sites possess a different protein composition from the rest of the fibrous membrane, containing a high concentration of globular proteins and proteoglycans [67]. Rodríguez-Navarro et al. [67] also reported that membranes obtained 7 h post-ovulation (initiation of eggshell mineralization) display weak staining with toluidine blue in the mammillary knob regions. In contrast, an intense blue staining is observed throughout the “bulk,” non-mineralized membrane fibers, indicating that mineralization only occurs at specific sites (Figure 4a) [67]. Since the outer membrane is partially mineralized, it constitutes the transition zone where the organic fibers merge and transform into the mineralized eggshell structure (Figure 4b–d). This transition zone has been described in detail [66]. The outer membrane contains discrete aggregates of organic matter intermixed with the fibrillar material and embedded into the mammillary knobs, which, if seen from the mineral columns, resemble an “opening flower bud”, also referred to as “bud-like structures.” These structures, also known as “mammillary cores, calcium reserve assembly, or mammillae” (from a mineralization point of view) [66,68,97], have been described as having a base plate that contains amorphous calcium carbonate and a “calcium reserve body” which are calcium crystals embedded in an organic core rich in sulfated proteoglycans. These organic aggregates are nucleation centers where the transition from amorphous calcium carbonate to calcite occurs (Figure 4) [67,100]. Specifically, at these organic aggregates, “mammillan”, a keratan sulfate proteoglycan, has been described, and its influence on calcium transport and mineral nucleation and formation has been proposed [100]. Thus, the ESM is critical for calcium carbonate crystallization during the initiation of eggshell mineralization, and these nucleation centers also offer the potential for directed calcium phosphate mineralization [101,102].

The ESM consists predominantly of proteins (around 80–85%) [1]. However, it also contains CaCO_3_ mineral, sialic acid, uronic acid, and a minimal quantity of polysaccharides [104]. In addition, small amounts of other ions, such as Mg, Si, and Zn, are also present [27,105]. The fibrous protein structure of the ESM is stabilized through extensive desmosine, isodesmosine, and disulfide cross-linkages, rendering it highly insoluble [91,105,106]. Almost 500 proteins have been identified in the ESM proteome, which consists of structural proteins (collagens, CREMPs) as well as globular proteins (ovocalyxin−36, lysozyme, lysyl oxidase, etc.) [91]. Lysozyme is abundant in the inner and limiting membrane; moreover, purified egg white lysozyme can induce changes in calcite crystal morphology in vitro [95]. The major structural protein is cysteine-rich eggshell membrane protein (abbreviated CREMP) [105], but it also contains 10% collagens (collagens I, V, and predominantly X). The outer and inner membranes have collagens I and X, but only the inner membrane possesses type V collagen [107]. According to estimates, the overall ratio between collagens I and V is 100:1 [108]. A small subset of proteins (#62) has been exclusively detected in the ESM of fertilized eggs at various stages of embryo development [109]. Comprehensive proteomic analyses of ESM have been performed [29,91,98,105,109,110,111]. The ESM possesses molecules similar to those of the bone matrix, namely type-I collagen in the fiber core and keratan sulfate in the mamillary knobs [1,100]. These molecules are essential in the nucleation processes of the shell mineral and might be useful for material development in bone tissue engineering.

The bulk elemental composition of the ESM is consistent, and no difference in the elemental composition of the different layers of the egg membrane has been detected [56]. EDS analyses have detected C (~47 wt.%), H (~6 wt.%), N (~15–27 wt.%), O (~12–22 wt.%), S (3 wt.%), and Ca (~0.42 wt.%) [56,72,76,112].

**Figure 5:**
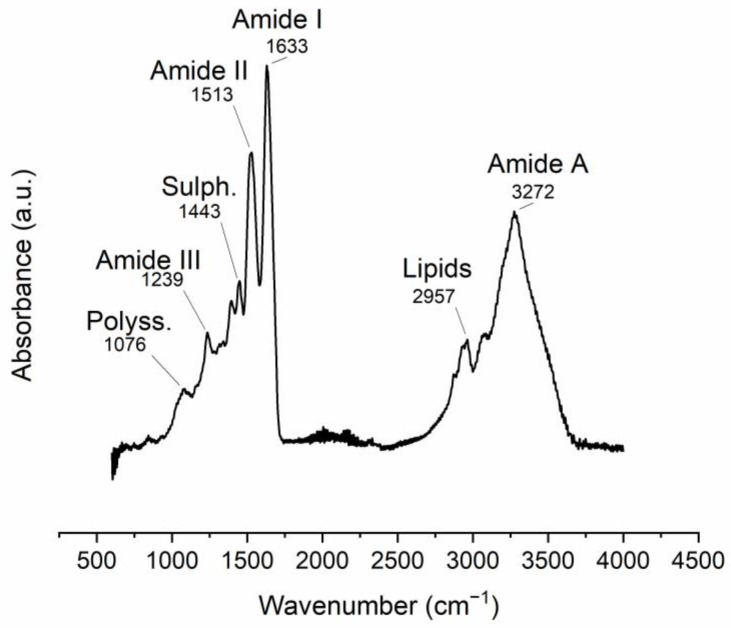
ATR-FTIR spectra of manually obtained ESM. Band position assignments are described in Table 3.

The ATR-FTIR spectra of the manually obtained ESM display vibrational bands related to its organic components. The most intense bands are related to protein-bond vibrations (Table 3, Figure 5).

**Figure 6:**
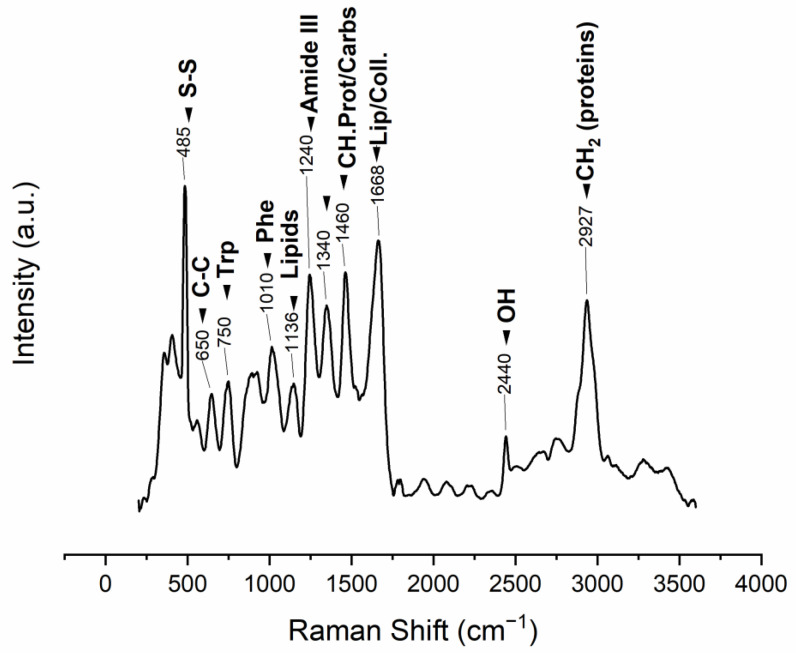
Raman vibrational bands observed in the manually obtained ESM (m: medium, s: strong, v: very, w: weak), band position assignments in Table 4.

The ESM Raman spectrum displays bands that can be associated with protein structure. The 485 cm^−1^ band is attributed to the sulfur-containing proteins (cysteine/disulfide-rich; CREMPs) of the membranes (Figure 6, Table 4).

**Figure 7:**
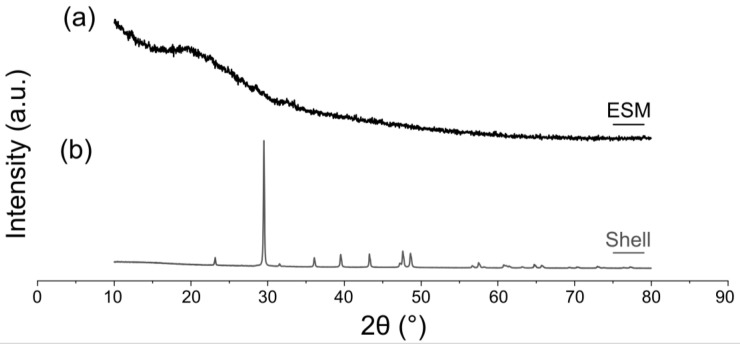
XRD patterns of the eggshell and ESM (mechanically removed from the shell). (**a**) ESM. (**b**) Mineral shell membrane without organic membrane. The membrane was removed manually.

The diffractogram of ESM, manually removed from the shell and air-dried on a flat support, is present in Figure 7. The X-ray diffraction (XRD) pattern of the ESM depicts its amorphous structure with a broad halo between 2θ = 10°–30°. (Figure 7a) This halo is attributed to the organic components of the membrane [63,67,76]. The organic ESM does not display any diffraction lines for calcite, compared with the pattern of the calcitic mineral shell without the organic membrane (Figure 7b).

## 5. Biological, Physical, and Mechanical Properties of the Eggshell Membrane

### 5.1. Biological Properties

One crucial aspect that bone tissue engineering scaffolds must possess is biocompatibility [124]. For this purpose, in vitro and in vivo assessments that include cellular, antibacterial, and animal tests are necessary to predict the behavior of a biomaterial in the human body. The biocompatibility of the ESM has been mostly explored in biomaterials based on the ESM or containing components obtained from it [125]. Therefore, there is still room to investigate the biological properties of the natural, unprocessed ESM. 

In this section, we have limited our focus to biological tests in which the membrane was modified minimally and used as a biomaterial. Modifications related to the membrane acquisition method were not considered (Table 5). Research on the egg membrane as a bone scaffold is slowly accumulating. Thus, to date, only a few studies have evaluated ESM for this purpose [53,107,108,109]. 

The ESM possesses good cytocompatibility, cell attachment, proliferation, and non-cytotoxicity. Cytocompatibility studies were performed by cell culture and MTT assays, supported by optical, scanning electron, and fluorescence microscopy techniques (Table 5). The main human cell types studied for biocompatibility include corneal mesenchymal stromal cells (C-MSC), human dermal fibroblasts (hDF), and cells derived from osteosarcoma. The corneal mesenchymal stem cells could attach to ESM and demonstrated non-exponential growth [56]. hDF cells proliferated after three days on the natural, untreated ESM, and the orange/ethidium bromide (AO/EB) double staining assay showed a low proportion of dead cells. Furthermore, these fibroblasts expressed human cytokines, such as monocyte chemoattractant protein-1 (MCP-1), growth interleukin-8 (IL-8), epidermal growth factor (EGF), regulated oncogene (GRO), and GRO-alpha (GRO-) when cultivated on either ESM or citric acid-modified ESM biomaterial. Additionally, the citric-acid-modified ESM-grown cells produced fewer of the pro-inflammatory cytokines IL-8 and MCP-1 and more of the pro-healing cytokines leptin and stem cell factor (SCF) [58]. 

In vivo studies with ESM have demonstrated its ability to improve tissue regeneration. Choi et al. [58] revisited and compared the natural ESM with an acid-modified ESM in injured-skin studies performed in rats. The natural ESM slightly improved healing compared to untreated injuries. The acid-modified ESM promoted the formation of a full-thickness epidermal layer with regenerated dermis and appendants, which accelerated wound healing. This interpretation was evidenced by a higher concentration of proliferating cells (Ki67-positive cells) and myofibroblast cells (SMA-positive cells) and a decrease in CD11b-positive immune cells, the latter of which were also present in natural ESM regenerated injuries. Modified ESM increased cell proliferation, wound contraction, angiogenesis, and regulation of inflammation. Processed ESM powder has been proposed as a promising biomaterial for tissue engineering [29]. It is the basis for an innovative wound-healing product [106,126], which is currently in clinical trials for treating venous leg ulcers (DermaRep^®^).

Implantation of ESM in bone has also been evaluated in rat calvaria and paravertebral defects, as well as rabbit ulna osteotomy [55]. These studies have been key for observing an inflammatory predisposition, bone resorption, or regeneration mechanism, as well as degradation of the membrane. In the case of rat calvaria defects, hydrolyzed, pepsin, and acid-treated ESM did not elicit bone resorption. Despite some deformation, the hydrolyzed ESM became tightly bonded to the skull and presented minimal to no inflammation, indicating its good biocompatibility [23]. The ESM was obtained by dissolving the shell mineral in acid, followed by ESM implantation in paravertebral regions. Arias et al. [55] observed that the primary healing mechanism in this model was via fibrosis. In contrast, the main healing mechanism in rabbit ulnar defects was via controlled bone bridging.

The ESM has been used in guided bone regeneration of critical-sized periodontal defects in Wistar rats, which is an in vivo model [57]. On one side of the mouth, the defects were filled with eggshell powder and covered with ESM. The eggshell-derived biomaterials did not elicit any foreign body or allergic reaction, and new bone formation was observed on both sides of the defect. Moreover, the inflammation did not show eosinophil infiltration. Kavartaphu et al. [57] concluded that the ESM prevented epithelial migration and encouraged osteoblast proliferation and angiogenesis. This study expanded the prospect of using this membrane for guided bone regeneration. However, in future research, the ESM could be combined with a xenograft commonly used in the clinical practice of guided bone regeneration, not only with eggshell powder. 

In vivo investigations are also essential for follow-up observations of the ESM degradation process. A degradable scaffold avoids the need for surgical implant removal [18,48]. Therefore, in the design of a scaffold, it is crucial to consider the degradability and breakdown process of a biomaterial [127]. The degradation rate of a bone scaffold must be harmonized to provide temporary mechanical support but degrade synchronously as the tissue regenerates. A relatively fast material breakdown cannot provide the structural support required for bone rebuilding [124]. Optimally, the degradation rate should resemble or be slower than the bone repair rate. Additionally, the by-products of its decomposition should be easily eliminated through normal physiological pathways without being toxic or harmful to the body. In the rat subcutaneous implantation model, ESM breakdown was reported after 16 weeks, whereas bone implantation in rabbit osteotomy varied from 8 to 16 weeks [55]. This is an important property to highlight because it could represent an advantage over other biopolymers, such as collagen membranes, which present unfavorable kinetics of degradation occurring between four days and six weeks after surgical placement [128]. The ESM can be effectively sterilized through autoclaving [61], treatment with ethanol [60], or ethylene oxide [55]. The performance of sterilized ESM has been tested in vivo in models that include chickens [60], rabbits, and rats [55]. However, it remains necessary to determine whether the ESM retains its primary structural and metabolic features following these sterilization processes. Other techniques, such as using UV-C light in conjunction with hydrogen peroxide (H_2_O_2_), have been employed with eggshell [129], although their impacts on the associated ESM were not studied.

### 5.2. Physical Properties

The complete thickness of the ESM, spanning its multilayered structure, is close to 0.1 mm (Table 6); some differences are related to the variables previously described. Despite the relative thinness of the membrane, it is easily manipulated for direct implantation [23] or further modification. Thickness is an essential characteristic since it has been linked to eggshell strength [130]. As previously described, the ESM physical and mechanical characteristics present dissimilarities due to breed, age, and the nutritional state of the hen [94]. Moreover, the variability of ESM thickness associated with developmental, morphometric, and environmental factors has also been described [131,132]. The thickness of the ESM is higher at the equator and decreases at the blunt and sharp poles of the egg. Lastly, eggs from hens exposed to pollutants (e.g., organochloride compounds) possess diminished ESM thickness and embryo survival [131]. In addition, Torres et al. [2] determined that water loss is an important factor affecting the ESM thickness via optical and atomic force microscopy measurements. Thus, additional research is warranted to explore the hypothesis that variations in fiber constituents as well as fiber size and distribution could influence this global physical property. 

The bone’s porous architecture influences the permeability and diffusivity of the materials. Porosity enables mass transportation and metabolic tissue activity [124]. Bone porosity varies according to the type of bone. Cortical bone is more compact and denser, having a lower degree of porosity than trabecular bone. Cortical bone porosity has been estimated as between 3% and 12%, while in trabecular bone, this varies between 50% and 90% [133]. Tortuosity and its influence on permeability is also strongly linked to porosity [50]. The ESM porosity ranges from 10% to 56% and is related to the humidity of the membrane and the type of test used (Table 6). Hsieh et al. [70] reported a pore dimension modification by treating the membrane with hydrogen peroxide (H_2_O_2_), which altered the pore size dimensions from 3–10 μm to 1–5 μm [70]. In this procedure, cysteine oxidation to cystine caused large-scale structural changes in the ESM [70]. Given the structural strength of the membrane, further studies could explore treatments to increase the pore size of the membrane and produce materials with an optimal range for bone growth of 100 to 130 μm [134]. Moreover, the ESM porous heterogeneous topography, pore size, and pore geometry are all factors that affect the tortuosity and could be tuned and modified to enhance the permeability, diffusivity, and mass transport desired for a membrane-based scaffold [50,135]. In applications requiring lower permeability with higher surface area, modification of the ESM porosity with H_2_O_2_ could be useful [70]. 

The chemical composition of the outer layer of the ESM controls its surface properties. These properties impact a variety of biological reactions that take place as a response to the biomaterial, including protein adsorption, cell adhesion and proliferation, and biocompatibility. Therefore, investigating the physicochemical parameters of the surface (e.g., surface charge, surface chemistry, roughness, and wettability) is essential to understanding the interfacial relationship between a biological system and the biomaterial [136]. 

Hydrophilicity constitutes a factor that affects the adherence and spreading of cells. Biocompatible surfaces are neither highly hydrophobic nor highly hydrophilic. Moderately wettable surfaces are considered more biocompatible, favoring a cellular response mediated by integrins [52,70]. Regarding this aspect, the membrane presents a different hydrophilic behavior on its inner and outer surfaces. This property is typically described with water angle contact measurements [58,59], around 40° for the inner membrane and between 40–70° for the outer membrane (Table 6). Hsieh et al. [70] found that treated and untreated ESM had a high contact angle of approximately 108° due to surface roughness. However, understanding the hydrophilic behavior of the membrane beyond a contact angle measurement could be helpful for material design. Surface treatments that modify the membrane hydrophilicity could enhance the biocompatibility of membrane-based materials, as has been done with other bone biomaterials [137]. Mensah et al. [56] approximated this behavior by describing the swelling and drying profile of the membrane. The rate of eggshell membrane swelling in PBS (phosphate-buffered saline) was rapid for the first 2 min and remained relatively constant after 10 min. On the other hand, during drying, the membranes experienced weight loss in the first three minutes and dried almost completely between 30 and 50 min.

**Table 6:** Outcome of physical properties measurements and mechanical tests performed on the ESM.

Property	Extraction	State	Method	Value	Ref.
**Thickness**	Manual	Humid	Micrometer	~0.096 mm	[56]
	Manual	Not specified	Micrometer	~0.080 mm	[138]
	Shell dissolution (acetic acid)			~0.124 mm	
	Shell dissolution (EDTA)			~0.122 mm	
	Manual removal	Dry	Confocal scanning laser microscopy	~50–70 μm (outer membrane)	[94]
				~15–26 μm (inner membrane)	
				~3.6 μm (limiting membrane)	
**Porosity**	Manual removal	Dry	SEM	56.54%	[56]
		Humid (ethanol)	Liquid displacement method	9.95%	[58]
		Humid (water)	AFM	52.06%	[2]
**Contact angle**	Manual removal	Dry	Contact angle meter. 2 μL PBS microdroplet	Between 40–50°	[56]
	Manual removal	Not described	Contact angle meter. 10 μL water microdroplet	~78°	[58]
	Shell dissolution (HCl)	Not specified	Drop shape analysis system goniometer. Water microdroplet	Inner membrane: 80.5° (3 s) 46.3° (2 min) Outer membrane: 99.8° (3 s), 68.8° (2 min)	[76]
**Burst strength**	Manual removal	Wet (PBS)	Texture analyzer	~2 N	[56]
**Tensile strength**	Manual removal	Wet (PBS)		0.9 MPa	[56]
	Manual removal	Not specified	Tensile testing machine	0.9–3.2 MPa	[138]
	Shell dissolution (acetic acid)	Wet	Texture analyzer	1.3 MPa	
	Shell dissolution (EDTA)	Wet		1.4 MPa	
	Manual removal	N.S	Universal testing machine	1.6 MPa	[58]
	Manual removal	N.S		1.6 MPa	[59]
	Manual removal	N.S	Tensile testing machine	6.4 MPa	[2]
	Manual removal	Wet (water)		1.4 MPa	
		Wet (albumen)		1.8 MPa	
**Young’s Modulus**	Manual removal	Wet		4.1 MPa	[56]
	Shell dissolution (acetic acid)	Wet (PBS)	Texture analyzer	3.3 MPa	
	Shell dissolution (EDTA)	Wet		3.6 MPa	
	Manual removal	N.S	Universal testing machine	~4.7–5.5 MPa	[59]
	Manual removal	Dry	Tensile testing machine	232 MPa	[2]
	Manual removal	Wet (water)		5.5 MPa	
		Wet (albumen)		5.3 MPa	

N.S; Not specified.

### 5.3. Mechanical Properties

Bone scaffolds are surgically implanted into a bone defect. The materials of which they are composed, such as ESM, will be subjected to various mechanical stresses. The biomaterials transmit these forces to the cell microenvironment, acting as biophysical cues for cells. Cells interpret these stimuli as chemical, physical, or biological signals. In turn, these signals influence their gene expression and regulate their cell behavior and function (phenotype) [124]. Therefore, the fabrication of bone scaffolds containing ESM that possess advantageous mechanical qualities for bone healing requires a basic understanding of the mechanical properties of the ESM.

The ESM, as well as most biopolymeric frameworks, possesses a viscoelastic mechanical behavior that reflects the dynamic nature of natural polymers and tissues. They are elastic, returning to their original shape when deformed, and viscous, with an unavoidable water component that resists fluidity [139]. The triple helical structure of collagen confers compressive and tensile strength to animal tissues and provides anchorage to cell adhesion via surface receptors [30]. The mechanical properties of the ESM have been found to be similar to those of other biopolymer materials [2,92]. As in tissues such as skin [140], cornea, tendon, and blood vessels, this behavior is determined by the behavior of individual fibers and the nature of the interactions between them [141]. They are heterogeneous and anisotropic, which makes them difficult to measure with the standardized mechanical tests typically used in material science [2,139]. Despite the high variability that biological materials exhibit, there have been various approaches to describing the mechanical behavior of ESM. 

Variations in the mechanical properties of the ESM may reflect the breed, age, living environment, and nutrition of the laying hen [94]. Mechanical test measurements will also differ if the membranes are dried or humidified in water, albumen, or other media [2]. In addition, the membrane extraction method can modify certain measurements (Table 6). 

The age of the laying hen egg affects the mechanical properties of the ESM [57,126]. According to Kemps et al. [142], the attachment force and breaking strength of the ESM decreased in eggs from hens in their early-to-mid-lay phase, but remained consistent after that. In addition, egg storage temperature is another factor considered a source of variability in ESM strength. However, although storage temperature significantly influences shell strength, it does not appear to impact the mechanical performance of the membrane [142].

Despite the high variability of the ESM anisotropic behavior, some of the main mechanical properties of the ESM related to its microstructure and strength have been compiled in Table 6. These properties will be described in the following sections. These static values comprise properties such as Young’s modulus, burst, and tensile strength and provide pertinent information on the ESM mechanical properties (Table 6). Still, it is necessary to consider the mechanical behavior of the ESM, which may evolve beyond a mere static value and could be the subject of study in future research.

Analysis of the mechanical behavior of a biomaterial will determine its performance for potential uses [2]. Collagen fibers form the extracellular matrix of connective tissues and are essential for tissue tensile strength [143]. Ultimate tensile strength (UTS) is defined as the maximum stress that a material can withstand while being stretched and can be determined by uniaxial tension tests of the extracted membranes. UTS is often associated with toughness, expressed as the energy absorbed by the ESM up to the breaking point per unit volume of the membrane [55]. Reported data have shown that fracture parameters, including UTS, fracture strain, and fracture toughness, increase with loading rate. Strnková et al. [138] evaluated the fracture parameters at 1, 10, 100, and 800 mm/min loading speeds. They found that, except for the lowest loading speed, all parameters increased regardless of the type of egg analyzed according to the commercial line (breed of chickens, geese, or Japanese quail). The lowest values of the fracture parameters were for quail ESM, while the highest were for goose ESM.

Biological materials exhibit mechanical property variability related to several factors, including sample heterogeneity, differences in cross-sectional measurements of the specimens used for the tests, and variations in biomaterial intrinsic moisture [2]. The tensile strength of chicken ESM exhibits a wide range from 0.9 to 6.4 MPa, which depends on its dry or wet state during these measurements (Table 6). Higher values are obtained when the membrane is in a dried state, as biopolymers are stiffer than in the hydrated state [144]. Minor differences have been detected for membranes in different hydration conditions, for example, in liquid albumen (0.2 MPa), which is the natural medium for this material [2]. This difference could be related to the hydrating properties of physiological solutions [144]. The main protein constituents of egg albumen (white) are ovalbumin (50–60%), ovotransferrin (15%), mucoid (8%), and lysozyme (2–3%) [145]. Egg albumen acts as a plasticizing agent for ESM fibers, similar to collagen-based tissues, which need to be hydrated to perform their normal biomechanical functions [2]. 

Young’s modulus is a measure of the stiffness of the material, and the values for ESM are highly variable, as is the case for other natural systems, such as collagen and animal tissues [101,146,147]. The variability in Young’s modulus values has been associated with the non-homogeneity of the biological tissues, especially with changes in the local humidity during the tests. The mechanical properties of ESM are highly dependent on the medium in which the biomaterial is tested, and Young’s modulus of ESM fibers could change because of dehydration during testing [2]. Torres et al. [2] reported that Young’s modulus values in chicken ESM ranged from 4.2 to 38.1 MPa and had a mean value of 19.8 ± 14.3 MPa. The dried state increases the number of hydrogen bonds in the protein chains, and the eggshell membrane is stiffer with a high Young’s modulus. However, when the membranes are plasticized by water or albumen, this value drops, reflecting the viscoelastic nature of this membrane. It possesses brittle behavior when dry, while this value decreases considerably in albumen and water [2]. Values from 0.25 to 3 GPa have been determined for pure type I collagen fibrils [140]. The differences in the results found for ESM can be related to the changes generated by the encapsulation of the collagen core by a layer rich in glycoproteins that acts as a template for the crystallization of calcium carbonate [30].

**Figure 8:**
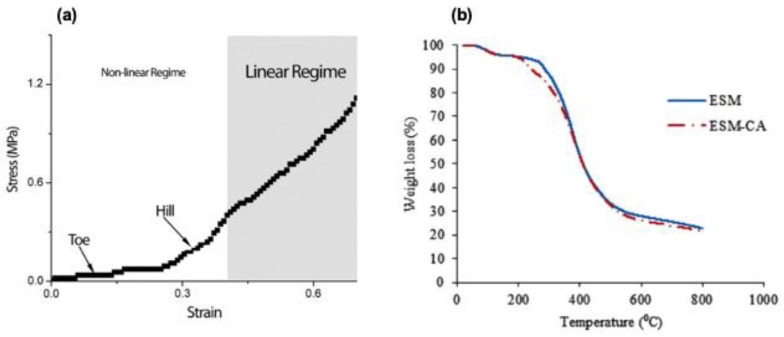
(**a**) Stress–strain curve representation for eggshell membrane. Image reproduced from Torres et al. [2] Copyright Elsevier (license number 5446550963203). (**b**) TGA curves for eggshell membrane and modified ESM. Figure reused from Gharibi et al. [59]. Copyright Elsevier (license number 5446560347938).

The ESM presents a stress–strain curve akin to other collagen-based systems, such as tendons. To deconvolute the complex elastic behavior of ESM, Torres et al. [2] described three regions (Figure 8a): toe, hill, and a region of linear dependency. In collagen-based systems, the toe region represents the “un-crimping” and the stretching of the fibers at a relatively low stiffness due to the entropic elasticity of the fibers [148]. The hill or heel region has been associated with increased stiffness as the membrane elongates, while the linear dependence region is where there is a proportional elongation response of the fibers to the load. Therefore, the membrane presents a non-linear regime at low load levels, where the measured stress and strain are not directly proportional, and a linear regime at higher levels, where the strain variation is directly proportional to the variation of effort [2]. 

The ESM has also been characterized by thermogravimetric analysis (TGA) (Figure 8b). The ESM TGA results display a multistep thermal decomposition pattern, with an initial change at around 50 ͦC, followed by a second stage at 130 °C, related to the beginning of collagen degradation and water loss. A dramatic third weight loss is associated with the thermal degradation of the protein structure, followed by a steady, continuous decrease until the complete decomposition of the ESM backbone [59]. The data from this analysis may provide a helpful baseline for comparison with ESM modified during biomaterial synthesis.

## 6. Calcium Phosphate Mineralization of the Eggshell Membrane

The mineralization of ESM with calcium phosphates, including apatite, has received little attention; however, the available research provides important details that may be helpful for the engineering of bone-like materials. Despite the relative paucity of articles, the heterogeneity of the methods of membrane extraction, pre-treatment, mineralization, and characterization yields a wide range of information on these aspects, and thus permits the selection and combination of the factors for the most effective membrane–apatite crystallization in future research. 

**Figure 9:**
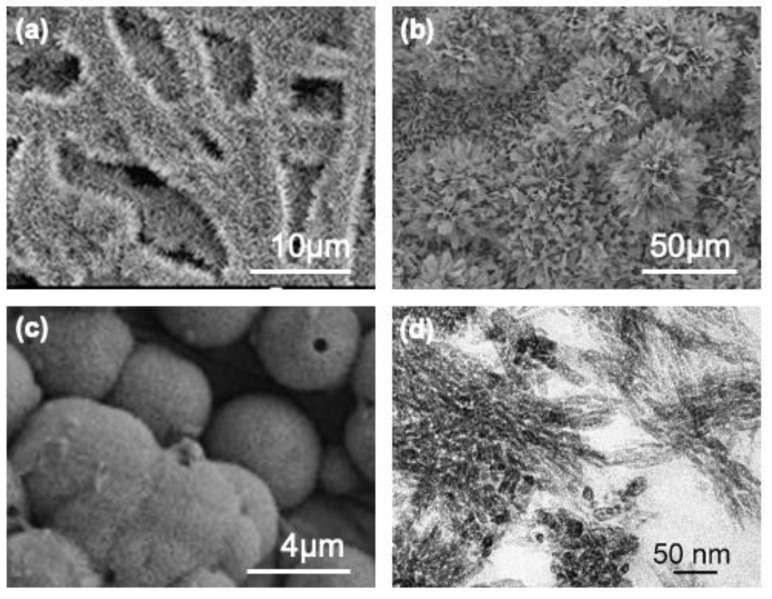
Morphologies of the deposits obtained in apatite mineralized eggshell membranes. (**a**) Needle-like apatite deposits. Image. Reproduced from Xu et al. [149]. Copyright Elsevier (License number 5471980705584). (**b**) Flower-like apatite aggregates. Image reproduced from Zhang et al. [101]. Copyright Elsevier (License number 5471980997435). (**c**) Globular-shaped mineral deposits. Image reproduced from Chen et al. [76]. Copyright Elsevier (License number 5471981207993). (**d**) Nanoplatelets in the core of the membrane fiber. Image reproduced from Li et al. [96]. Copyright Elsevier (License number 5471981394702).

Four studies that successfully report mineralizing the ESM with apatite are compared here (Table 7). These studies have confirmed the nucleating ability of ESM through various approaches. The methods employed to mineralize the membrane primarily involve soaking the membrane in buffered solutions (HEPES or simulated body fluid (SBF)) or a combination of calcium and phosphate solutions. Intriguingly, Zhang et al. [101] carried out membrane mineralization by employing the ESM as a diffusion membrane separating calcium and phosphate solutions. Additionally, they examined the mineralization at various intervals, from 3 days to 4 weeks (Table 7). Crystals were grown with different morphologies, including needle-like, globular, and flower-like crystals and nanoplatelets (Figure 9). Nevertheless, only two studies report mechanical testing results on the mineralized membranes [96,149], and only a single study performed biological testing [76] (Figure 9).

The mineralized membranes possess enhanced mechanical properties [96,149] and improved biological activity [76] compared to the unmineralized ESM. The mineralized membranes displayed increased hardness and reduced Young’s modulus. Xu et al. [149] describe the membranes as having higher microhardness values compared to controls. In the study by Li et al. [96], the membrane was mineralized with apatite and silica, so the observed increase in the hardness and Young’s modulus cannot be attributed merely to apatite incorporation. Regarding biocompatibility, the apatite mineralized membranes prepared by Chen et al. [76] exhibited increased cellular attachment, proliferation, and expression of osteogenic proteins compared to the unmineralized ESM.

Another difference between the studies was how the membrane was obtained and treated before mineralization. In two of these studies, the membrane was retrieved manually [96,101], while in the other two, the shell was dissolved in HCl to recover the membrane [76,149]. In two articles, the membrane underwent pre-treatment with enzymes, acid, and the addition of polyanionic solutions before mineralization [96,150]. In contrast, in the other studies, the membrane had minimal pre-treatment [76,101]. The method of membrane extraction is an overlooked but important step because chemical solutions can negatively modify the protein membrane structure [78]. Similarly, the pre-treatment with pepsin enzyme has been discouraged as it severely digests the membrane [149]. Conversely, the treatment with polyanionic solutions could enhance mineralization. These substances introduce anionic phosphate groups into the membrane, which simulate the nucleating function of phosphorylated non-collagenous proteins in vivo [96]. These solutions, including sodium trimetaphosphate (SMTP), also modified the morphology of theobserved mineral deposits. SMTP pre-treated membranes presented needle-like deposits, while without SMPT, plate-like shapes were obtained (Table 7).

Zhang et al. [101] explored variables that modify apatite precipitation on the ESM, namely temperature, pH, incubation time, and specifying the surface of the membrane tested. This last aspect is relevant since in vivo, the inner surface of the inner ESM is in contact with the albumen, while the external surface is in contact with the mineralizing eggshell. They observed that at higher temperatures and pH, the driving force toward nucleation increased, with enhanced crystallinity of the resulting precipitate. Mineralization at higher temperatures and pH tended to yield mainly hydroxyapatite. In contrast, the membranes mineralized at lower temperatures and pH exhibited a mixture of calcium hydrogen phosphate phases and apatite. Nevertheless, the sizes of the mineral deposits tended to be smaller at higher pH and temperature. In this investigation, the incubation time was also a key aspect [101]. At shorter times, the kinetics of ionic incorporation into the apatite lattice is inadequate, while longer experimental times are associated with the dissolution of apatite and the reprecipitation of calcium hydrogen phosphate hydrate. Both sides of the membrane were visualized during the mineralization process in this study [101]. In normal eggshell calcification, the fibers at the outer surface of the membrane are associated with ACC precipitation, calcite nucleation, and crystal growth [67]; therefore, it was expected that this surface would display preferential mineralization. However, mineral deposits were also detected on the inner surface of the membrane. When comparing the deposits that developed on the two faces, the deposits on the inner face tended to be smaller. However, to fully assess this aspect, more data are necessary. Chen et al. [76] also studied both sides of the membrane and performed biological tests with MC3T3-E1 preosteoblastic mouse cells. Their experiments revealed that cells associated with fibers of the inner membrane displayed higher cell proliferation, differentiation, and expression of osteogenic proteins, including Run-x, alkaline phosphatase, and osteocalcin. They attributed this behavior to increased smoothness of the inner surface (reduced nanotexture).

According to these findings, the ESM can be calcified with apatite using relatively straightforward procedures, which enhance its mechanical and biological characteristics. Certain factors must be considered for apatite precipitation, including the method of obtaining the membrane, treatment prior to mineralization, the active surface of the membrane, pH, temperature, and experimental duration. However, the development of ESM-apatite composite materials is still an emerging field. Other precipitation methodologies remain to be investigated, as well as further mechanical characterization tests and in vitro and in vivo biocompatibility tests.

## 7. Limitations of the Present Review

The biological, physical, and mechanical properties (as a whole) of the pristine unmodified ESM were considered in order to assess its potential either as biomaterial by itself or as a bi-dimensional scaffold to build future bone regenerative materials. The information available in the current literature has been summarized from this perspective, and indicates that, in principle, the properties of the ESM meet some of the requirements for bone tissue engineering scaffolds. However, the limited nature of the data available prevents a deeper and more extensive evaluation. Thus, this review identifies gaps and needs that will encourage further research with the aim of exploiting the ESM in the field of bone tissue engineering and other areas that could repurpose this biomaterial for biomedical applications.

## 8. Concluding Remarks and Future Perspectives

This review has underlined the biological, physical, and mechanical properties of the ESM that render it suitable for bone biomaterial development. The ESM’s inherent nucleating ability, its compositional resemblance to the extracellular bone matrix, and the simplicity of its mineralization with calcium phosphate phases, including apatite, all suggest that it is suitable for the development of hybrid materials. The calcium-phosphate-mineralized ESM imitates the dualistic nature of bone, wherein the ESM is the organic polymeric part, and the calcium phosphate mineral is the ceramic-like inorganic part; its exploitation could be further explored in bone tissue engineering. The ESM is a basis for designing multiscale scaffolds that reproduce hierarchical bone structure from the nano- to the macroscale. This can be achieved by combining the ESM with other materials that could provide a microscopical and gradient pore-sized structure. The macroscale properties of these constructs could favor osseointegration and angiogenesis, while the nano- and microscale properties of the membrane provide osteoconductivity [151]. For example, particalized eggshell membrane (PEM) may provide a format suitable for 3D printing of ESM–based bone tissue engineering scaffolds that correspond to the size and shape of the bony defect [150]. Moreover, the ESM constitutes an economical and widely available resource, and its sustainable reuse would contribute to the circular economy. This approach could also reduce manufacturing costs compared to other, more expensive bone regenerative materials that require extensive processing. There is still a long way to go, from optimizing the membrane extraction process and selecting/tailoring the mineralizing methodology to characterizing and testing the ESM in vitro and in vivo. However, we hope this review encourages interest in this versatile biopolymer and promotes consideration of its use and application in bone regeneration.

## Figures and Tables

**Table 1:** Eggshell chemical dissolution approaches to obtain ESM.

Acid	Concentration	Soaking Time	Temperature	Ref.
Acetic Acid	70% *w/w*	Two days	N.S ^1^	[72]
	2 % *w/w*	30 min	N.S	[74]
	1%	10 min ^2^	N.S	[55]
	0.5 M	44 h	RT ^3^	[56]
HCl	1 M	N. S	N. S	[65]
	0.03 M	10 min	~100 °C	[75]
	1 M	One hour	25 °C	[76]
EDTA	5% *w/w*	One day	N.S	[77]
*n*-butyl acetate	5% *w/w*	30 min	RT	[74]

^1^ N.S: Not specified. ^2^ The eggs were emptied and filled with the acid solution, not soaked. ^3^ RT: Room temperature.

**Table 2:** Different ESM separation methodologies indicating their pros and cons.

Method	Pros	Cons
Manual peeling	No chemical alteration of the ESM Possible modification of structural ESM attachments [29].	Time-consuming Difficulty in obtaining large-sized pieces [78].
Chemical dissolution	Larges pieces of ESM can be obtained.	Alteration of the organic structure [78]. Lack of an established protocol with optimized chemical concentration, time, and temperature [78]. Contamination of the environment by the generated aqueous waste [82].
Microwave detachment	Use of a conventional microwave oven. Differential heating ESM/eggshell.	Possible alteration in the ESM biological and physicochemical properties due to thermal stress/heating [81].
Flash evaporation [82].	Simple equipment. Low energy consumption Good separation rate	Unknown effect in the ESM structure
Mechanical appliances	Suitable for industrial-scale use.	Air pollution caused by dust [81] Subject to intellectual property protection (patents)
Enzymatic method	Alternative method.	Complex reaction conditions Costly production (e.g., expensive proteases) [73,82].

**Table 3:** FTIR vibrational bands observed in the ESM (m: medium, s: strong, v: very, w: weak), table based on [58,60,63,70,96,113,114,115].

Band Position (cm^−1^)	Intensity	Vibration Description
1076	w	(Attributed to polysaccharides)
1239	w	Amine C-N stretching (Amide III)
1443	w	CH_2_ scissoring (attributed to sulfates)
1513	s	C-N stretching/NH bending (Amide II)
1633	vs	Amide C=O stretching (Amide I)
2426	vw	Sulfhydryl group (-SH) ^1^
2957	m	C-H stretching (attributed to lipids)
3272	S	O-H and N-H stretching (Amide A)

^1^ possibly due to proteins rich in cysteine (i.e., CREMPS).

**Table 4:** Raman vibrational bands observed in the ESM (cm^−1^; m medium, s strong, v very, w weak); table based on the assignments in [116,117,118,119,120,121,122,123].

Band Position (cm^−1^)	Intensity	Vibration Description
485	vs	ν(S–S) stretching vibration
650	m	Tyrosine and phenylalanine C-C twisting mode
750	m	Symmetric breathing of tryptophan
1010	m	Phenyl ring angular bending vibrations, related to phenylalanine
1136	s	Lipids
1240	s	Amide III
1340	s	CH deformation (proteins and carbohydrates)
1460	s	CH_2_ wagging, CH_2_/CH_3_ deformation for lipids and collagen
1668	vs	Amide I
2440	w	OH stretching vibrations
2927	s	CH_2_ asymmetric stretch

**Table 5:** Biological tests performed with eggshell membrane. The cellular and animal tests are described.

**Cellular Tests**					
**Test**	**Cell Tested**	**Biomaterial**	**Method**	**Time Span**	**Ref.**
Cytotoxicity, cell attachment, and cell proliferation.	Corneal mesenchymal stromal cells (C-MSC)	Untreated natural ESM for corneal wound healing	Cell culture	1, 3, and 7 days	[56]
	Human dermal fibroblasts (hDF) (GIBCO and C0135C).	Untreated, natural ESM, and ESM treated with acetic and citric acid	MTT assay	1, 2, and 3 days	[58]
	Osteosarcoma fibroblast-like MG-63	Modified ESM with citric acid for drug delivery systems and tissue engineering	MTT assay	1 and 2 days	[59]
**Animal Testing**					
**Test**	**Animal Tested**	**Biomaterial**	**Method**	**Time Span**	**Ref.**
In vivo skinwound healing	Male Sprague-Dawley rats (7-weeks-old, weighing 200–230 g)	Untreated, natural ESM, and ESM treated with acetic and citric acid	10 mm skin injuries. Histological and immunohistochemical evaluation	0, 3, 7, and 10 days	[58]
Bone regeneration membrane	Wistar rats	Hydrolyzed ESM treated with pepsin and acetic acid as a membrane for guided bone regeneration	6 mm calvaria defects. Radiographical and histological examination	60 days	[23]
Subcutaneous implantation	Sprague-Dawley white rats	Acid removed and sterilized ESM as an anti-bone bridging membrane	Paravertebral implantation	1, 2, 4, 6, and 16 weeks	[55]
Anti-bone bridging implantation	New Zealand white rabbits	Acid removed and sterilized ESM as an anti-bone bridging membrane	Rabbit ostectomy and implantation	Between 8 and 16 weeks	[55]
Guided bone regeneration membrane	Wistar rats	Untreated, natural ESM for guided bone regeneration.	Periodontal defect performed filled with eggshell powder and covered with ESM. Histological observations	45 days	[57]

**Table 7:** Eggshell membrane calcium phosphate mineralization studies.

Membrane Preparation	Membrane Pre-Treatment	Mineralization Procedure	Time Span	Mineral Detected	Method to Identify the Mineral	Mineral Characteristics	Mechanical Tests	Biological Tests	Ref.
Shell dissolution (HCl)	Pepsin, SMTP (also without SMTP), Ca(OH)_2_	HEPES solution incubation	1–4 weeks	Without SMTP: amorphous calcium phosphate. SMTP: hydroxyapatite	XRD, FTIR	Without SMTP: plate-like crystals. SMTP: needle-like crystals.	Microhardness	None.	[149]
Manual extraction	None	Membrane placed as a barrier between K_2_HPO_4_ and calcium acetate.	3–12 days	Mixture of calcium hydrogen phosphate and hydroxyapatite, crystalline hydroxyapatite	XRD, TEM	Flower-like crystals.	None	None	[101]
Shell dissolution (HCl)	None	1.5 SBF incubation	1–7 days	Hydroxyapatite	XRD, FTIR	Globular	None	Cell culture on MC3T3-E1 mouse- pre-osteoblasts. ALP assay, osteogenesis-related-gene protein expression assay. Western blot.	[76]
Manual extraction	3-mercaptopropionic acid, acetic acid, STPP	Incubation in CaCl_2_, K_2_HPO_4_, HEPES, and polyacrylic acid	14 or 28 days.	Calcium phosphate, apatite, silica nanoparticles.	TEM, FTIR	Nanoplatelets	Nanoindentation.	None.	[96]

Abbreviations: SMTP, sodium trimetaphosphate; STPP, sodium tripolyphosphate; XRD, X-ray diffraction; FTIR, Fourier transformed infrared spectroscopy; TEM, Transmission electron microscopy.

## Data Availability

Not applicable.
